# Investment Trait, Activity Engagement, and Age: Independent Effects on Cognitive Ability

**DOI:** 10.1155/2012/949837

**Published:** 2012-06-25

**Authors:** Sophie von Stumm

**Affiliations:** Department of Psychology, University of Edinburgh, 7 George Square, EH8 9JZ Edinburgh, UK

## Abstract

In cognitive aging research, the “engagement hypothesis” suggests that the participation in cognitively demanding activities helps maintain better cognitive performance in later life. In differential psychology, the “investment” theory proclaims that age differences in cognition are influenced by personality traits that determine when, where, and how people invest their ability. Although both models follow similar theoretical rationales, they differ in their emphasis of behavior (i.e., activity engagement) versus predisposition (i.e., investment trait). The current study compared a cognitive activity engagement scale (i.e., frequency of participation) with an investment trait scale (i.e., need for cognition) and tested their relationship with age differences in cognition in 200 British adults. Age was negatively associated with fluid and positively with crystallized ability but had no relationship with need for cognition and activity engagement. Need for cognition was positively related to activity engagement and cognitive performance; activity engagement, however, was not associated with cognitive ability. Thus, age differences in cognitive ability were largely independent of engagement and investment.

## 1. Introduction

In cognitive aging research, the “engagement hypothesis” predicts that engagement in physical, social, and intellectual activity contributes to reducing age-related cognitive decline and the risk of neurodegenerative disorders [1, 2]. That is, frequent participation in cognitively demanding activities is thought to “exercise” the brain with more cognitively engaged people having better cognition over time because of practice benefits. Thus, the preservation of cognition is thought to depend on the extent to which “a diverse behavioral repertoire is integrated into daily life” [3, page 487]. In differential psychology, the “investment theory” suggests that age-related changes in cognitive development are influenced by personality traits that determine where, when, and how people apply their mental ability [4, 5]. Thus, investment traits are thought to predispose individuals to seek cognitively stimulating environments that in turn prompt the development, application, and practice of cognitive strategies [3, 5]. That said, investment traits may also lead to approaching even mundane experiences in a cognitively stimulating manner, thereby enhancing intellectual development (cf. [6]).

In spite of their native disciplines' differential emphasis on decline versus growth, the engagement hypothesis and investment theory have a lot in common. First, both models propose that individual differences in intellectual engagement are reflected in lifespan trajectories of cognitive development [1, 5]. Second, both models have received some empirical support (e.g., [7–9]), as well as some rejections (e.g., [10–12]). Third, both are subject to the same criticism that the effects of engagement or investment on cognitive change (i.e., differential preservation) are explained by alternative factors, in particular by prior cognitive ability (i.e., preserved differentiation, cf. [2]). That said, the engagement hypothesis and investment theory also differ in one crucial point: cognitive aging researchers tend to assess differences in engaging in substantively complex environments, while investment theorists measure latent traits of personality that refer to “the tendency to seek out, engage in, enjoy, and continuously pursue opportunities for effortful cognitive activity” [13, page 225]. That is, activity engagement is typically assessed with reference to a specific set of activities or environments, such as going to the theatre, while investment traits refer to the intrinsic motivation to think, and corresponding scales assess, for example, one's preference of complex over simple problems. Despite following different rationales, investment and engagement measures rely equally on self-reports, and neither construct has a gold standard scale or equivalent (cf. [2, 13]). Cognitive aging measures of activity engagement vary in their foci, ranging between the frequency of an activity (e.g., regular versus sporadic; [14]), its intensity (e.g., gentle versus vigorous exercise; [15]), life-stage-specific activities (e.g., educational attainment in young adulthood versus occupational achievements in later life; [16]), and specific activity domains (e.g., social versus physical; [11]). Conversely, theoretical and psychometric definitions of investment range from comparatively narrow investment trait scales (e.g., need for cognition; [17]) to broad trait dimensions (e.g., openness to experience; [18]), to even broader trait complexes [5]. To systematically address the role of engagement and investment for cognitive performance, the current study compares the need for cognition scale and a measure of cognitive activity engagement, as well as their relationship with age differences in cognitive ability.

Two previous studies that assessed a wide range of activities, including, for example, housework and religious service attendance, found little support for the notion that activity engagement mediated the effects of an investment trait on cognitive performance [3, 19], which may have been due to the breadth of the included investment and engagement measures. Here, a narrowly focused scale was developed to assess the frequency of participating in typical cognitive activities (e.g., reading a novel; visiting a museum). To measure individual differences in intellectual investment, the need for cognition scale was selected. It refers to the “tendency to engage in and enjoy thinking” [17, page 116] and is a widely used, well-validated and precise measure of investment (cf. [20–22]). Need for cognition scale items makes no reference to specific cognitive activities or environments but measure the extent to which a person enjoys deliberating, abstract thinking and problem solving [17].

In line with previous research [3, 23], it was hypothesized that need for cognition was not meaningfully associated with age because it is a relatively stable trait dimension. Conversely, the frequency of activity engagements is likely to change according to age. That is, during some life periods that allow for the time and financial resources (e.g., young adulthood or early retirement), activity levels can be expected to be relatively high compared to others that are more restricted (e.g., adolescence and parenthood). It follows that activity engagement may have a nonlinear relationship with age.

**Figure 1:**
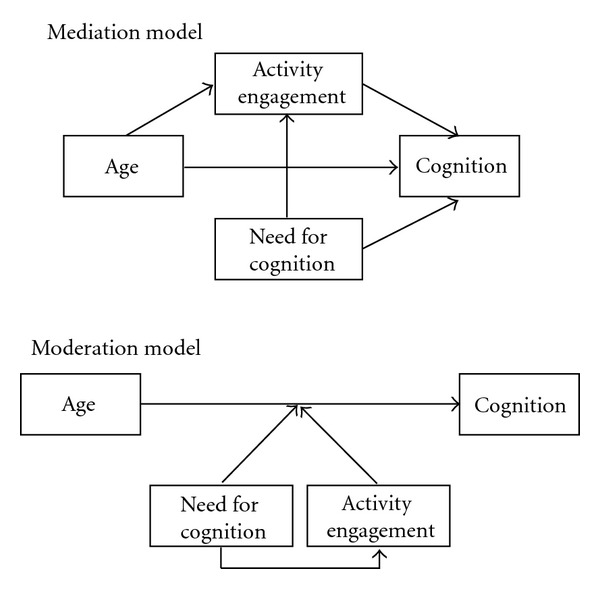
Mediation and moderation models.

With respect to age differences in cognitive ability, the so-called fluid abilities (i.e., reasoning capacity) were expected to be negatively correlated with age, while the crystallized abilities (i.e., vocabulary) were expected to be positively associated with age (cf. [4, 5]). Accordingly, age differences in fluid and crystallized ability may be mediated or moderated by cognitive activity engagement and need for cognition (cf. [24, 25]; Figure 1). In a mediation model, the effect of age on cognition is accounted for by activity engagement, which in turn should be positively associated with need for cognition. Thus, the predisposition to seek cognitively stimulating environments is thought to result in a greater frequency of activity engagement, which explains part of the association between age and cognition. By comparison in a moderation model, strength and direction of the relationship between age and cognition is expected to depend on the level of activity engagement and need for cognition (cf. [24]). Thus, people with high need for cognition and subsequently frequent cognitive activity engagements may show smaller age differences in fluid ability and greater ones in crystallized ability than those with low need for cognition and few activity engagements. Because mediation and moderation models are equally plausible in this research context, the current study explores both alternatives.

## 2. Methods

### 2.1. Sample

200 British adults (97 men) were recruited with an average age of 34.6 years (SD = 11.8; range from 18 to 69 years; two participants did not report their age). As their highest educational qualification, 14% participants had completed general certificates of secondary education (10th grade); 15% A-levels (12th grade); 18% a vocational qualification or equivalent; 33.5% an undergraduate degree, and 19% a postgraduate degree. About half of the sample reported to earn less than *£*15.000 ($22,500) per annum, while about 8% declared to earn more than *£*35.000 ($52,000) per annum.

### 2.2. Measures

**Need for Cognition (see [17])**

The 18-item scale measures the desire to engage in effortful cognitive activity on a 5-point Likert scale, ranging from strongly disagree, disagree, somewhat agree, agree, to strongly agree. An example item reads: “I would prefer difficult to simple problems.” Internal consistency typically ranges from  .83 to  .97 [20].

**Cognitive Activity Engagement**

Nine items that were most frequently used in previous studies to assess cognitive activity engagement were adapted [7, 14, 26] and complemented by one addressing the use of modern information technology (i.e., google; Table 1). Participants indicated on a Likert-type scale how often they engaged in the activities listed ranging from 1 to 5, including never, once or twice a month, every week, every other day, and every day.

**Cognitive Ability**

Fluid and crystallized abilities were assessed with three tests each, including Raven's matrices [27] and five other tests [28]. *Fluid ability:* (1) Raven's progressive matrices: 12 items showed grids of 3 rows × 3 columns, each with the lower right-hand entry missing. Participants chose from 8 alternatives the one that completed the 3 × 3 matrix figure. The test was timed at 4 minutes. (2) Lettersets: in 5 sets of 4 letters, participants identified the set that did not fit a rule that explained the composition of the other 4 lettersets. The test had 15 items and was timed at 6 minutes. (3) Nonsense syllogisms: participants judged if a conclusion that followed two preceding statements (premises) showed good (correct) reasoning or not. The test had 15 items and was timed at 4 minutes. *Crystallized ability:* (1) verbal reasoning: participants had to identify the correct pair of words from five options to complete a comparison sentence, whose first and last works were missing. The test had 14 items and was timed at 7 minutes. (2) Vocabulary: participants had to identify the correct synonym for a given word out of five answer options. The test had 18 items and was timed at 4 minutes. (3) Verbal fluency: participants had to write down as many words as possible that started with the prefixes “sub” and “pro.” For each prefix, 60 seconds were allowed.

### 2.3. Procedure

Participants were recruited in London, England, with online and flyer advertisement. Inclusion criteria were as follows: native English speakers; normal or corrected to normal vision, hearing, and motor coordination; having lived in the United Kingdom for at least 10 years. These criteria were self-reported by the participants prior to testing. No university students were recruited. Participants completed a two-hour testing session in groups of up to twenty in designated research laboratories. The ability tests were administered in 40 minutes, then participants completed a range of other measures (not reported here), and finally, they completed the cognitive activity engagement and need for cognition scale, as well as a demographic background questionnaire in their own time (approximately 15 minutes). They received monetary compensation.

### 2.4. Analysis

The intelligence tests' *z-*scores were added to form unit-weighted composite scores of fluid and crystallized ability. The cognitive activity responses were weighted on a linear frequency scale of days per annum (i.e., every day = 365; every other day = 182; every week = 52; once or twice a month = 18; never = 0); the psychometric properties of the scale were subsequently analyzed. The study variables were investigated for sex differences in means and variances, and then, their intercorrelations were computed. Next, a series of path models tested if age differences in cognition were mediated or moderated by need for cognition and cognitive activity engagement. To test for mediation, a path model was fitted in line with Figure 1, including fluid and crystallized ability as correlated outcome variables. To test for moderation, all variables were *z*-transformed. A series of regression models tested two-way interactions (need for cognition × age, activity engagement × age, and need for cognition × activity engagement) and a three-way interaction (age × need for cognition × activity engagement) separately for fluid and crystallized ability. That is, a first set of models (one for fluid, one for crystallized ability) included age and need for cognition in a first step and in a second, their interaction term. A second set of models included age and activity engagement in a first step and then their interaction. A third set of models included first age, activity engagement, and need for cognition, next their two-way interactions, and finally the three-way interaction term.

## 3. Results

Table 1 shows the descriptives for the cognitive activity engagement items after recoding the Likert scale into days per annum. Item endorsement frequencies did not vary meaningfully with age. A unit-weighted composite score was formed; the corresponding coefficient alpha was  .58. The activity engagement score was normally distributed, and so were the test scores of all cognitive ability tests. No meaningful sex differences were observed in the study variables, and thus, data from men and women were analyzed together.

The scatterplot suggested that age was not associated with cognitive activity engagement, neither in a linear nor in a nonlinear fashion.  Table 2 shows the descriptives of and correlations between age (in years), need for cognition, activity engagement, and fluid and crystallized ability. Age was significantly negatively associated with fluid and positively with crystallized ability. Furthermore, fluid and crystallized ability were intercorrelated (*r* = .66), and so were cognitive activity engagement and need for cognition, albeit to a much smaller extent (*r* = .25). Cognitive activity engagement was not correlated with age, fluid or crystallized ability, while need for cognition had a significant, positive associations ability but not with age.

**Figure 2:**
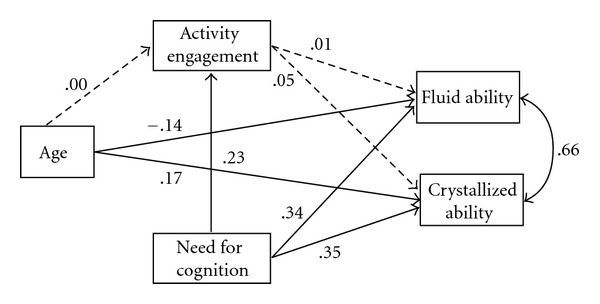
Mediation model of age differences in cognitive ability with standardized path parameters. Note: error terms for activity engagement, fluid and crystallized ability have been omitted to sustain graphical clarity. Dashed paths represent nonsignificant pathways (*P* > .05). The double-headed arrow indicates a correlation.

Figure 2 shows the mediation model results. As before, age was positively associated with crystallized ability and negatively with fluid intelligence, while need for cognition had positive relationships with activity engagement, fluid and crystallized ability. Activity engagement did not mediate any of the age or need for cognition effects on cognitive ability. Thus, need for cognition and age had only direct effects on fluid and crystallized ability, accounting for 13% and 15% of their total variance, respectively.

Table 3 shows the results of the moderation models. In the first step, age was positively associated with crystallized and negatively with fluid ability, while need for cognition was positively associated with both, and activity engagement was not meaningfully related to ability. Neither two-way nor three-way interaction yielded any significant results. Thus, the level of activity engagement or investment did not interact with age differences in fluid and crystallized abilities. Overall, the results suggest that while investment traits and cognitive activity engagement are moderately associated, neither affects age differences in cognition. That said, need for cognition was significantly correlated with cognitive ability, while activity engagement was not.

## 4. Discussion

 The current study explored the relationship of an investment personality trait (i.e., need for cognition) and a cognitive activity engagement scale with age differences in cognitive performance. In line with earlier research [3, 19], need for cognition and cognitive activity engagement were positively interrelated, albeit weakly so. Therefore, a predisposition to deliberate and think abstractly is somewhat different to actively pursuing cognitively stimulating engagement, such as reading a novel or going to the theatre. Also consistent with previous findings [1, 5], age was negatively associated with fluid and positively with crystallized ability, as well as unrelated to the investment trait need for cognition (cf. [17, 23]). Contradicting the current hypotheses, however, no meaningful age differences were observed in cognitive activity engagement. Thus, while the frequencies of activity engagement were slightly elevated in age groups that are likely to experience the most advantageous conditions for engagement (i.e., financial security and time), these differences were not significant.

Confirming previous research [20, 21, 29], need for cognition was positively associated with both fluid and crystallized ability, while no such association was observed for cognitive activity engagement (cf. [3, 19]). Furthermore, cognitive activity engagement did not mediate the association between age and cognition. That is, age and need for cognition had direct, independent effects on cognition, which were unrelated to cognitive activity engagement. It seems plausible that need for cognition contributes to constructing everyday experiences in an intellectually enriching way, and thus, the effect of need for cognition on cognitive performance is direct and not mediated by engagement (cf. [3, 6]). Future research must establish how need for cognition affects perception and perhaps even intellectual exploitation of daily working and living routines, and how such experiences contribute to cognitive development and aging.

The current study has several limitations. First, the study design was cross-sectional, and all causal inferences are speculative. Second, the recruitment methods of the study may have led to a biased sample composition by attracting particularly active or cognitively engaged individuals. Also, the age range of participants (18 to 69 years), about half of whom were aged between 18 and 30 years, is possibly not ideal for detecting age differences in cognition. Indeed, the modesty of the observed associations between age and cognition is likely to be due to the relative youth of the current sample. The latter is unlikely, however, to account for the observed zero-order associations of age with investment and engagement because the sample spanned several life periods. Third, the current study assessed only one dimension of activity engagement (i.e., cognitive), but it may be that other engagement aspects, such as physical or social activity, are more important for age differences in cognition [7]. Also, only the frequency of cognitive activity engagement but not its duration nor the complexity of the activity was assessed here. Finally, the assessment instruments of intellectual investment and cognitive activity both relied on self-reports, which are known to be influenced by social desirability and self-serving bias (cf. [30]).

Notwithstanding these shortcomings, the current study contributes to understanding the role of investment and engagement for age differences in cognition. Echoing previous research (e.g., [7, 9, 11, 12]), it seems as if intellectual engagement—regardless of being assessed in terms of activity participation or trait disposition—has little effect on age differences in cognition. That said, the predisposition to invest (i.e., need for cognition) in one's cognitive competence contributed overall to better cognitive performance and a higher frequency of cognitive activity engagement (cf. [3, 9]). To explain the relationship between investment and cognition, mechanisms other than activity engagement must be explored, for example, individual differences in constructing experiences within daily living routines.

## Figures and Tables

**Table 1:** Descriptives of cognitive activity engagement items.

	Item	*N*	*M*	SD
1	Read a book?	198	135.13	141.54
2	Read the newspapers?	199	203.59	137.46
3	Attend a music event or concert?	199	29.38	56.88
4	Attend evening classes?	197	18.71	43.91
5	Write for pleasure?	200	63.16	109.54
6	See a play at the theatre?	197	17.76	37.32
7	Go to a museum or gallery?	198	29.45	44.59
8	Attend a public talk or lecture?	196	22.03	50.23
9	Visit the cinema?	199	35.70	64.12
10	Google things?	200	273.56	126.51

Note: activity engagement was recorded on a 5-point scale and recoded in days per annum (i.e., every day = 365; every other day = 182; every week = 52; once or twice a month = 18; never = 0).

**Table 2:** Correlations and descriptives for study variables.

		*N*	*M*	SD	1	2	3	4
1	Fluid ability	200	0.00	2.31	—			
2	Crystallized ability	189	−0.01	2.52	.66^∗^	—		
3	Age (years)	198	34.58	11.84	−.14^∗^	.18^∗^	—	
4	Activity engagement	193	830.32	357.84	.08	.10	.00	—
5	Need for cognition	189	3.46	0.60	.34^∗^	.35^∗^	.00	.25^∗^

*P < .05.

Note: need for cognition was recorded on a 5-point Likert scale, ranging from strongly disagree, disagree, somewhat agree, agree, to strongly agree. Activity engagement was also recorded on a 5-point scale and recoded in days per annum (i.e., every day = 365; every other day = 182; every week = 52; once or twice a month = 18; never = 0).

**Table 3:** Standardized regression parameters for two-way and three-way moderation models.

Step	Model 1						Model 2						Model 3					
	Fluid ability			Crystallized ability			Fluid ability			Crystallized ability			Fluid ability			Crystallized ability		
	*β*	CI (95%)		*β*	CI (95%)		*β*	CI (95%)		*β*	CI (95%)		*β*	CI (95%)		*β*	CI (95%)	
1																		
Age	**−.14**	**−0.63**	**−0.01**	**.17**	**0.08**	**0.77**	−.11	−0.59	0.07	**.19**	**0.12**	**0.86**	−.13	−0.63	0.03	**.19**	**0.12**	**0.86**
NFC	**.34**	**0.44**	**1.05**	**.35**	**0.51**	**1.16**	—	—	—	—	—	—	**.32**	**0.39**	**1.02**	**.35**	**0.50**	**1.18**
CAE	—	—	—	—	—	—	.07	−0.18	0.48	.09	−0.13	0.59	.03	−0.27	0.38	.05	−0.22	0.48
2																		
NFC × age	−.03	−0.37	0.24	−.04	−0.41	0.24	—	—	—	—	—	—	−.05	−0.43	0.23	−.01	−0.38	0.32
CAE × age	—	—	—	—	—	—	−.05	−0.43	0.20	−.05	−0.45	0.24	−.05	−0.46	0.23	.01	−0.36	0.39
CAE × NFC	—	—	—	—	—	—	—	—	—	—	—	—	−.09	−0.43	0.10	.00	−0.28	0.29
3																		
CAE × NFC × age	—	—	—	—	—	—	—	—	—	—	—	—	.09	−0.13	0.36	.13	−0.07	0.45

Note: the regressions were stepwise conducted, entering the respective set of independent variables in step 1, and their corresponding interaction terms in step 2 and 3. All models were run separately for fluid and crystallized ability as dependent variables. Model 1 shows the results for the two-way interaction of age and need for cognition; model 2 shows the two-way interaction of age and cognitive activity engagement; model 3 shows the results of testing for the three-way interaction. Significant parameters are shown in bold. Keys: age = age in years; NFC = need for cognition; CAE = cognitive activity engagement.
